# Impairment of endothelial function in Parkinson’s disease

**DOI:** 10.1186/s13104-022-06176-z

**Published:** 2022-09-05

**Authors:** Branislav Kollár, Andrej Blaho, Katarína Valovičová, Michal Poddaný, Peter Valkovič, Igor Straka, Peter Turčáni, Pavel Šiarnik

**Affiliations:** 1 1st Department of Neurology, Faculty of Medicine, Comenius University, Mickiewiczova 13, 813 69 Bratislava, Slovakia; 2 AB Neuro, Jilemnickeho 547/10, 911 01 Trencin, Slovakia; 3 Department of Neurology, General Hospital, Palucanska 25, 031 23 Liptovsky Mikulas, Slovakia; 4 2nd Department of Neurology, Faculty of Medicine, Comenius University, Limbova 5, 83305 Bratislava, Slovakia; 5 Institute of Normal and Pathological Physiology, Centre of Experimental Medicine, Slovak Academy of Sciences, Sienkiewiczova 1, 813 71 Bratislava, Slovakia

**Keywords:** Atherosclerosis, Dopamine agonist, Endothelial function, Parkinson’s disease

## Abstract

**Objective:**

There are conflicting data regarding the relationship between Parkinson’s disease (PD) and the atherosclerotic process. This study aimed to compare endothelial function in patients with PD and matched controls. In PD subjects, we searched for factors contributing to endothelial dysfunction as well. Traditional vascular risk factors, PD characteristics, and PD medication were considered.

**Results:**

We prospectively enrolled 41 patients with PD and 41 controls matched for age, sex, body mass index, and vascular risk factors. Endothelial function (EF) was assessed using peripheral arterial tonometry (EndoPAT 2000 device) and expressed as reperfusion hyperemia index (RHI). Clinical characteristics including PD medication were recorded. RHI was non-significantly lower in the PD group than in controls (1.8 ± 0.5 vs. 1.9 ± 0.5, p = 0.478). In PD patients, in linear regression analysis, smoking (beta = −0.453, p = 0.008) and use of dopamine agonists (beta = -0.365, p = 0.030) were significant contributors in a model predicting RHI. Despite non-significant differences in endothelial dysfunction between PD patients and controls, our results suggest an association between smoking, dopamine agonists, and impaired EF in PD patients. The small sample size, as well as the absence of an extended search for traditional and non-traditional vascular risk factors, are the most important factors limiting the interpretation of the current results.

## Introduction

Parkinson’s disease (PD) belongs to the most common neurodegenerative diseases. Resting tremor, rigidity, bradykinesia, and postural instability belong among classical motor symptoms of PD. PD is accompanied by the loss of dopaminergic neurons and Lewy pathology. Possible underlying mechanisms include oxidative stress, mitochondrial dysfunction, diminished neurotransmitter level, and perturbed protein homeostasis [1]. PD patients suffer from a variety of comorbidities including complications of atherosclerotic diseases [2]. There are conflicting data regarding the relationship between PD and the atherosclerotic process. Some studies have found a higher incidence of vascular disease in PD patients, while others described their similar or even lower incidence in comparison to the matched non-PD population [3–6]. Likewise, studies of the subclinical atherosclerotic process provide inconsistent results. Using the measurement of carotid intima-media thickness (IMT), some authors concluded that PD patients have a lower risk of atherosclerosis, while others reported that the IMT in PD patients was significantly higher than in controls [7–9]. Endothelial dysfunction (ED) is an initial process and a key component of atherogenesis [10]. Previous studies indicated that endothelial function (EF), as assessed by the flow-mediated dilation (FMD), was significantly decreased in PD patients [11, 12]. Several possible mechanisms are explaining the impairment of EF in PD. Both PD and endothelial dysfunction possibly share multiple underlying mechanisms including mitochondrial dysfunction and oxidative stress [13]. Additionally, many of the well-established cardiovascular risk factors promote atherogenesis. These include non-modifiable risk factors such as age, sex, and positive family history, as well as modifiable ones. Traditional vascular risk factors include smoking, hypertension, dyslipidemia, and diabetes mellitus. However, mechanisms are not always completely understood and multiple non-traditional vascular risk factors may play a role [14]. PD patients with L-dopa therapy exhibit increased levels of homocysteine. Hyperhomocysteinemia can lead to vascular disease [15]. Although the use of dopamine agonists might be associated with cardiovascular complications, their impact on EF has not been studied so far [16]. Both, FMD of the brachial artery and peripheral arterial tonometry (PAT) of digital arteries are non-invasive measures of endothelial function that assess vascular endothelium dilation in response to shear stress forces. FMD is the more widely used technique. However, PAT is a promising method offering the advantage of easier use, and relative operator independence [17]. To the best of our knowledge, this is the first study to investigate EF using PAT in PD patients. In this study, we aimed to compare EF in patients with PD and controls matched for age, sex, and traditional vascular risk factors. Impaired EF in PD subjects was suspected. In PD subjects, we additionally searched for factors contributing to endothelial dysfunction as well. Traditional vascular risk factors, PD characteristics, and PD medication were considered.

## Main text

### Methods

Study subjects were recruited from patients attending an Outpatient Clinic of the 1^st^ Department of Neurology and the 2nd Department of Neurology, University Hospital in Bratislava, Slovakia. PD patients fulfilled the UK Brain Bank Criteria for idiopathic Parkinson’s disease. Patients with clinical signs of vascular parkinsonism, parkinsonian plus syndromes, and PD dementia were excluded [18, 19]. Clinical and demographic characteristics including age, sex, body mass index (BMI), medical history, duration of disease, and PD medication were recorded. The modified Hoehn–Yahr scale (H&Y) was used for staging PD while patients were in the ‘‘on’’ state [20]. The control population consisted of patients without PD, who were recruited from participants of our previous studies [21, 22]. Controls matched to the PD patients by age, sex, and traditional vascular risk factors (including the history of hypertension, diabetes mellitus, coronary heart disease, dyslipidemia, and smoking habit). Our study was approved by the Institutional Review Boards and patients provided informed consent before testing.

EF was assessed using PAT (EndoPAT 2000 device, Itamar Medical, Caesarea, Israel) and expressed as a reperfusion hyperemia index (RHI) as previously described [21]. The values of RHI below 1.67 were considered as ED [23].

The statistical analyses were performed using SPSS version 25 (SPSS Inc., Chicago, USA). Continuous variables were expressed as means ± standard deviation or median, interquartile range (IQR), minimal and maximal values, categorical variables as numbers, and proportions (%). To compare groups, Student's t-test, Mann–Whitney test, and Chi-squared test were used for particular variables. Pearson or Spearman correlation coefficients were used to determine the relationships between RHI and baseline characteristics. Stepwise multiple linear regression was used to create the prediction model and identify the most important contributors to this model. A model including the highest number of significant predictors was chosen. The dependent variable in the model was RHI. In the model, we included the following baseline characteristics as independent variables: age, gender, BMI, presence of arterial hypertension, coronary artery disease, diabetes mellitus, dyslipidemia, smoking, orthostatic hypotension, H&Y, duration of PD, use of l-dopa, levodopa equivalent dose (LED), use of catechol-O-methyl transferase (COMT) inhibitors, dopamine agonists, monoamine oxidase B (MAO-B) inhibitors, and use of amantadine. Each model was assessed for the presence of multicollinearity of included variables. Variance inflation factors (VIF) ≥ 5 were indicative of multicollinearity. P values < 0.05 were considered statistically significant.

## Results

The study population consisted of 41 PD patients and 41 controls matched for age, sex, BMI, and traditional vascular risk factors. Characteristics of the studied subjects are shown in Table 1. RHI was non-significantly lower in the PD group than in controls (1.8 ± 0.5 vs. 1.9 ± 0.5, p = 0.478). Presence of ED was more frequent (but not significant) in PD patients compared to controls (46.3% vs. 34.1%, p = 0.260).

**Table 1:** Characteristics of the study population

	Parkinsonʼs disease	Controls	p-value
Number of subjects	41	41	
Male/female sex	24/17 (58.5%/41.5%)	24/17 (58.5%/41.5%)	1.0
Age (years)	65.4 ± 9.1	65.3 ± 10.7	0.947
BMI (kg/m^2^)	27.5 ± 4.8	27.5 ± 3.6	0.996
Arterial hypertension	20 (48.8%)	21 (51.2%)	0.825
Coronary heart disease	6 (14.6%)	6 (14.6%)	1.0
Diabetes mellitus	8 (19.5%)	7 (17.1%)	0.775
Dyslipidaemia	6 (14.6%)	8 (19.5%)	0.557
Smoking	6 (14.6%)	6 (14.6%)	1.0
RHI	1.8 ± 0.5	1.9 ± 0.5	0.478
Endothelial dysfunction	19 (46.3%)	14 (34.1%)	0.260
Hoehn and Yahr score	0, 2.5 (0–4.0)	–	–
Duration of PD since diagnosis (years)	0, 5.0 (0–15.0)	–	–
LED (mg)	900.0 (0–2280.0)	–	–
L-dopa	31 (75.6)	–	–
Dopamine agonists	25 (61%)	–	–
COMT inhibitors	18 (43.9)	–	–
MAO-B inhibitors	14 (34.1)	–	–
Amantadine	6 (14.6%)	–	–

*PD* Parkinsonʼs disease, *BMI* body mass index, *RHI* reperfusion hyperemia index, *PB* blood pressure, *LED* levodopa equivalent dose, *COMT* catechol-O-methyl transferase, *MAO-B* monoamine oxidase

In PD patients, we failed to find any significant correlation of RHI with age, BMI, H&Y, duration of the disease or LED, see Table 2. Similarly, among PD patients, RHI did not significantly differ between males and females (1.8 ± 0.5 vs. 1.8 ± 0.6, p = 0.700), in subjects with hypertension (1.7 ± 0.5 vs. 1.8 ± 0.6, p = 0.545), coronary artery disease (1.6 ± 0.5 vs. 1.8 ± 0.5, p = 0.450), diabetes mellitus (1.9 ± 0.6 vs. 1.8 ± 0.5, p = 0.605), dyslipidemia (1.5 ± 0.6 vs. 1.8 ± 0.5, p = 0.148), smoking habit (1.4 ± 0.4 vs. 1.8 ± 0.5, p = 0.084). No significant difference was found in subjects with postural hypotension (1.5 ± 0.4 vs. 1.8 ± 0.5, p = 0.149), using L-dopa (1.8 ± 0.5 vs. 1.6 ± 0.6, p = 0.327), dopamine agonists (1.7 ± 0.6 vs. 1.9 ± 0.5, p = 0.208), COMT inhibitors (1.9 ± 0.5 vs. 1.7 ± 0.6, p = 0.433), MAO-B inhibitors (1.9 ± 0.6 vs. 1.7 ± 0.5, p = 0.444), and amantadine (1.7 ± 0.7 vs. 1.8 ± 0.5, p = 0.533).

**Table 2:** Correlations between reperfusion hyperemia index (RHI) and baseline characteristics of the PD subjects

Variable	r-value	p-value
Age	0.105	0.514
Body mass index	−0.095	0.556
Hoehn and Yahr scale	0.027	0.873
Duration of PD	0.190	0.245
LED	−0.083	0.618

*PD* Parkinsonʼs disease; *RHI* reperfusion hyperemia index; *LED* levodopa equivalent dose

In PD patients, the model predicting RHI in stepwise multiple linear regression analysis had R^2^ = 0.255, p = 0.030. Smoking (beta = −0.453, p = 0.008) and use of dopamine agonist (beta = −0.365, p = 0.030) were significant contributors in this model. VIF of all variables assessed in this model were ˂ 5.

## Discussion

Our data suggest no significant ED in patients with PD when compared to controls matched for traditional vascular risk factors. Values of RHI in the PD group were non-significantly lower than in controls and ED was non-significantly more frequent in PD patients compared to controls. In PD patients, according to linear regression analysis, smoking and the use of dopamine agonists were significant contributors in the model predicting RHI.

ED is an initial process and a key component of atherogenesis [10]. To the best of our knowledge, there have been only two previous studies regarding the EF in PD patients and controls, both using flow-mediated dilation (FMD). Our study is the first that used peripheral arterial tonometry (PAT). Our results are consistent with FMD studies, showing lower RHI in PD subjects compared to controls [11, 12].

Several possible mechanisms are explaining the impairment of EF in PD. l-dopa therapy exhibits increased levels of homocysteine that is considered a modest independent risk factor for vascular disease, causing endothelial damage and atherogenesis [15, 24]. Yoon et al. concluded that EF, as assessed by the FMD, may be associated with chronic l-dopa treatment in patients with PD [11]. Our results suggest no significant association between RHI and l-dopa treatment. Absence of the homocysteine testing is a limitation of our study. However, it is not clear whether hyperhomocysteinemia is a cause or just an epiphenomenon of ED [25]. Similarly, elevated plasma homocysteine was found in PD patients, particularly in those under l-dopa treatment, and there was no correlation between homocysteine and certain markers of endothelial dysfunction [26].

Dopamine agonists can be also involved in the process of atherogenesis. Their use could be associated with cardiovascular complications including heart failure and orthostatic hypotension (OH) [16]. Patients with OH can develop supine hypertension and fluctuations in blood pressure associated with OH can also contribute to atherogenesis [27]. Repeated bouts of hypertension and non-dipping patterns of hypertension may lead to shear stress and consequent endothelial damage [28, 29]. Also in prospective studies, the presence of OH was associated with an increased risk of subsequent vascular disease [30, 31]. The use of dopamine agonists is also associated with significant elevations in systolic blood pressure and heart rate, which can be involved in the development of ED [16]. Our results suggest that the use of dopamine agonists belongs among significant contributors in a model predicting RHI. OH was non-significantly more frequent in subjects using dopamine agonists compared to the rest of PD patients (24% vs. 12.5%, p = 0.365). However, potential mechanisms linking the use of dopamine agonists in PD patients should be elucidated by future prospective studies.

Except for dopamine agonists, smoking was another significant contributor in the model predicting RHI. The association of smoking with atherosclerosis is well-known and smoking belongs among traditional vascular risk factors [14].

Despite no significant difference in EF between PD patients and controls, pathomechanisms underlying PD could be also involved in atherogenesis. Oxidative stress is a well-known phenomenon associated with pathogenic mechanisms of several diseases including atherosclerosis, neurodegenerative disorders, and aging processes [32]. Both PD and atherosclerosis possibly share multiple underlying pathomechanisms including mitochondrial dysfunction and oxidative stress. Several substances affecting these mechanisms (including Mucuna pruriens, ursolic acid, and chlorogenic acid) demonstrated the neuroprotective effect in the Parkinsonian mice model [33–35]. However, future studies are needed to demonstrate the atheroprotective effect of such substances.

## Conclusion

Our data suggest no significant impairment of EF in PD patients compared to controls matched by age, sex, and traditional vascular risk factors. However, our study included “real world” PD patients suffering from multiple comorbidities. The Association of PD with ED should be explored more closely in populations with a lower burden of vascular risk factors to elucidate possible links between neurodegeneration and atherosclerosis. It could also help to explore the potential atherogenic effects of PD medications.

## Limitations

Except for the relatively small sample size, we have to admit several other limitations. Impairment of EF in PD patients can be influenced by multiple other underlying mechanisms, including traditional and non-traditional vascular risk factors. Smoking, a well-known traditional risk factor, was identified as a significant contributor in a model predicting RHI in this study. However, several non-traditional risk factors could be also involved in atherogenesis in the PD population. Physical inactivity, sleep disorders, or even vitamin D deficiency are common in PD patients and all of them can be associated with atherogenesis [12, 21, 36, 37]. The lack of search for non-traditional risk factors, including atherogenic amino acids testing, belongs to the limitations of a current study, and their role should be considered in future studies.

## Data Availability

The datasets used and/or analyzed during the current study are available from the corresponding author on reasonable request.

## Abbreviations

COMT: Catechol-O-methyl transferase
ED: Endothelial dysfunction
FMD: Flow-mediated dilation
H&Y: Hoehn–Yahr scale
IMT: Carotid intima-media thickness
IQR: Interquartile range
LED: Levodopa equivalent dose
MAO-B: Monoamine oxidase B
OH: Orthostatic hypotension
PAT: Peripheral arterial tonometry
PD: Parkinson’s disease
RHI: Reperfusion hyperemia index
VIF: Variance inflation factors
